# Holothurian triterpene glycoside cucumarioside A_2_-2 induces macrophages activation and polarization in cancer immunotherapy

**DOI:** 10.1186/s12935-023-03141-z

**Published:** 2023-11-24

**Authors:** Wen-Han Chuang, Evgeny Pislyagin, Liang-Yu Lin, Ekaterina Menchinskaya, Oleg Chernikov, Valery Kozhemyako, Tatiana Gorpenchenko, Igor Manzhulo, Elena Chaikina, Irina Agafonova, Alexandra Silchenko, Sergey Avilov, Valentin Stonik, Shey-Cherng Tzou, Dmitry Aminin, Yun-Ming Wang

**Affiliations:** 1 Department of Biological Science and Technology, Institute of Molecular Medicine and Bioengineering, College of Biological Science and Technology, National Yang Ming Chiao Tung University, Hsinchu, 300 Taiwan; 2 Center for Intelligent Drug Systems and Smart Bio-devices (IDS²B), National Yang Ming Chiao Tung University, Hsinchu, 300 Taiwan; 3 Far Eastern Branch, G.B. Elyakov Pacific Institute of Bioorganic Chemistry, Russian Academy of Science, 159, Pr. 100 let Vladivostoku, Vladivostok, 690022 Russia; 4 Pacific State Medical University, Ostryakova Avenue, Building 2, Vladivostok, 690002 Russia; 5 Federal Scientific Center of East Asia Terrestrial Biodiversity, Far Eastern Branch of the Russian Academy of Science, 159, Pr. 100 let Vladivostoku, Vladivostok, 690022 Russia; 6 A.V. Zhirmunsky National Scientific Center of Marine Biology, Far Eastern Branch of the Russian Academy of Science, Palchevskogo str. 17, Vladivostok, 690041 Russia; 7 Department of Biomedical Science and Environmental Biology, Kaohsiung Medical University, No. 100, Shin-Chuan 1st Road, Sanmin District, Kaohsiung City, 80708 Taiwan

**Keywords:** Holothurian triterpene glycoside, Cucumarioside A_2_-2, Anticancer, M1 macrophage, Immunotherapy

## Abstract

**Background:**

Despite intensive developments of adoptive T cell and NK cell therapies, the efficacy against solid tumors remains elusive. Our study demonstrates that macrophage-based cell therapy could be a potent therapeutic option against solid tumors.

**Methods:**

To this end, we determine the effect of a natural triterpene glycoside, cucumarioside A_2_-2 (CA_2_-2), on the polarization of mouse macrophages into the M1 phenotype, and explore the antitumor activity of the polarized macrophage. The polarization of CA_2_-2-pretreated macrophages was analyzed by flow cytometry and confocal imaging. The anti-cancer activity of CA_2_-2 macrophages was evaluated against 4T1 breast cancer cells and EAC cells in vitro and syngeneic mouse model in vivo.

**Results:**

Incubation of murine macrophages with CA_2_-2 led to polarization into the M1 phenotype, and the CA_2_-2-pretreated macrophages could selectively target and kill various types of cancer in vitro. Notably, loading near-infrared (NIR) fluorochrome-labeled nanoparticles, MnMEIO-mPEG-CyTE777, into macrophages substantiated that M1 macrophages can target and penetrate tumor tissues in vivo efficiently.

**Conclusion:**

In this study, CA_2_-2-polarized M1 macrophages significantly attenuated tumor growth and prolonged mice survival in the syngeneic mouse models. Therefore, ex vivo CA_2_-2 activation of mouse macrophages can serve as a useful model for subsequent antitumor cellular immunotherapy developments.

## Background

Cell-targeted immunotherapy, based on the activation and delivery of cells with antitumor activity directly to the tumor target in the body, is one of the newest and most rapidly developed approaches for cancer treatments. Active cell therapy usually involves isolation of immune cells from the blood or tumors of the patient. These tumor-specific cells are cultured, activated, and injected into the patient, where they begin to attack the tumor. The types of cells used in this way are natural killer cells (NK-cells), cytotoxic T cells, dendritic cells, and macrophages, including engineered cells and microrobots [1–8]. Unlike T-cells, whose use in cancer immunotherapy has serious limitations since these cells show limited capacity to enter the tumors [9, 10], macrophages can extricate from the bloodstream and move to the infiltrate cancer cells [11]. Therefore, macrophages are one of the immune cells best suited for targeted cellular immunotherapy, and the use of macrophages from the patient himself ensures no rejection due to MHC.

The phenotype of macrophages is quite plastic and malleable, and if macrophages are properly stimulated and reoriented, they can become reliable and effective mediators in the fight against tumors due to their ability to kill malignant cells, suppress angiogenesis, and reduce fibrosis [12]. Macrophages can be activated and polarized with immunomodulators to enhance their antitumor properties [13]. There are several functional states of polarization of macrophages, and they can be completely polarized and acquire a specific phenotype, such as M1 (classically activated macrophages) or M2 (alternatively activated macrophages). Macrophages of M1 phenotype can recognize and destroy neoplastic cells in vitro and in vivo, while non-tumor cells are unaffected. Although the exact mechanism(s) by which M1 macrophages distinguish tumor cells from normal cells is not yet fully understood, activated and properly polarized macrophages have great potential for cancer immunotherapy [14, 15].

Macrophages can be polarized by a number of molecules that cause them to differentiate into different functional types. In laboratory practice, classically activated M1 macrophages are usually obtained by incubation with LPS or LPS in combination with IFN-γ. This process is accompanied by the appearance of specific markers on the surface of macrophages, an increase in the secretion of pro-inflammatory cytokines, and other agents that enhance the inflammatory response and antitumor activity of macrophages [16–24]. However, due to the toxicity and uncontrollable effects of endotoxin LPS on cells, these inducers are inapplicable in clinical practice. In these regards, searching for safe compounds capable of activating macrophages and enhancing their tumoricidal activity is an urgent task.

A natural triterpene glycoside, cucumarioside A_2_-2 (CA_2_-2), isolated from the Far-Eastern edible sea cucumber *Cucumaria japonica* [25] has been found to have pronounced immunomodulatory effects in vitro and in vivo. Glycoside accelerates macrophage adhesion, spreading, mobility, and proliferation. Glycoside also increases lysosomal activity and enhances the phagocytosis and bactericidal ability of leukocytes by activating oxygen-dependent killing mechanisms. The compound induces the production of cytokines tumor necrosis factor (TNF-α), IL-6, and IFN-γ by human mononuclear cells. Glycoside could reactivate the expression of membrane-associated proteins CD3, CD4, and CD8 in the lymphocyte pretreated with the immunosuppression compound hydrocortisone. CA_2_-2 modulated the expressions of several intracellular proteins in mouse splenocytes. The most significantly affected proteins are NSFL1 cofactor p47, hnRNP K, Septin-2, NADH dehydrogenase [ubiquinone] iron-sulfur protein 3, and GRB2-related adapter protein 2, which are directly involved in the regulation of the activity of immune cells [26–31]. The pharmacokinetic study showed that intraperitoneal injection of glycoside rapidly accumulates and absorbs in the mouse spleen tissue at high concentrations, resulting in changes in the ratio of red to white pulp of the organ and increasing the proliferative activity in the white pulp. Moreover, marked changes in the protein/peptide profile have been observed as representative of exposure to immunostimulants. CA_2_-2 stimulation is accompanied by increased macrophage activating markers iba-1, IL-1β, ROS, iNOS, and NO, depicting the change of splenic macrophage phenotype to M1 [32–34]. Thus, CA_2_-2 is a promising agent for macrophage activation for antitumor cellular immunotherapy.

In recent years, nanoparticles (NPs) have become an important tool for various applications in nanomedicine due to their nanoscale nature. Owing to relatively uncomplicated synthesis, low cost, low toxicity, and the ability to modify the structure and composition of the surface, superparamagnetic iron oxide NPs are widely applied in various biomedical research, such as molecular imaging, stem cell tracking, tumor tissue targeting, drug delivery, hyperthermia treatment, and tissue repair [35]. The fluorescent nanoparticles are applied as the functional probes for cancer targeting and real-time monitoring of the drug release. The materials with NIR fluorescence can display low background signals and penetrate deep into the tissues, which is useful for broad in vivo clinical applications [36].

**Fig. 1:**
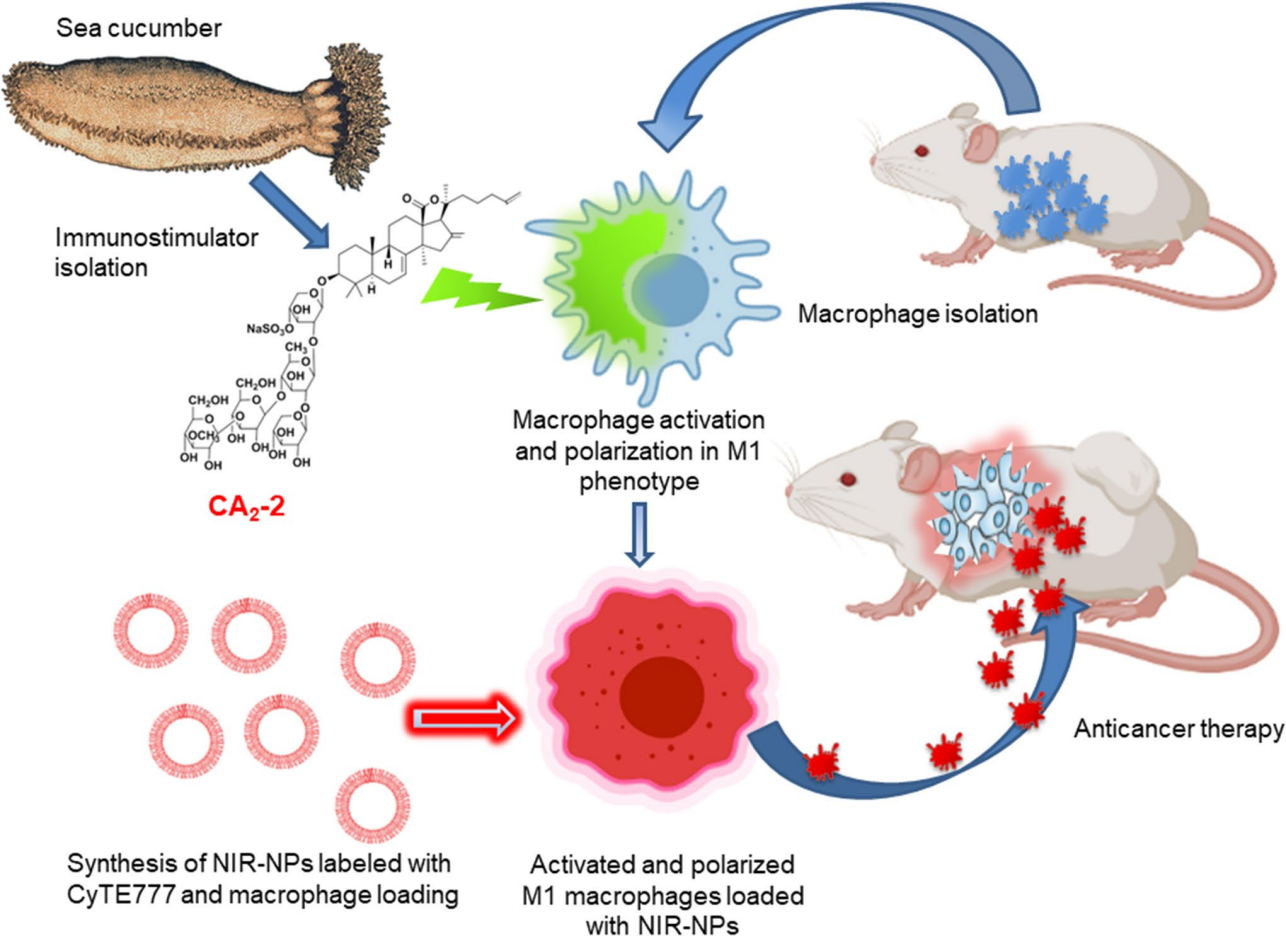
Strategy of cellular anticancer immunotherapy. Mouse macrophages were activated with sea cucumber triterpene glycoside cucumarioside A_2_-2 (CA_2_-2) and polarized in M1 tumoricidal phenotype ex vivo. Then, macrophages were loaded with synthesized nanoparticles conjugated with near-infrared fluorescent probe CyTE777 (NIR-NPs). After that, activated and polarized fluorescent M1 macrophages were injected into tumor bearing mice for macrophage visualization and localization and for anticancer cellular immunotherapy

In this work, we studied the effect of cucumarioside A_2_-2 on the activation and polarization of mouse macrophage cells into M1 phenotype ex vivo, and investigated the possibility of using CA_2_-2-activated and polarized M1 macrophages for antitumor cellular immunotherapy in mice with various types of tumors. Synthesized NPs conjugated with NIR fluorescent probe were loaded into macrophages for in vivo cell visualization and localization. The overall experimental strategy is illustrated in Fig. 1.

## Materials and methods

### Reagents

3-(4,5-dimethylthiazol-2-yl)-2,5-diphenyltetrazolium bromide (MTT, M2128), lipopolysaccharide (LPS, L3129), Ammonium persulfate (APS, 2300-OP), potassium hexacyanoferrate (II) trihydrate (P3289), and 2′,7′-Dichlorofluorescin diacetate (DCF2DA, D6883) were purchased from Sigma-Aldrich (St. Louis, MO, USA). The primary antibody (CD86-FITC and MHCII-RPE Cy5, ARG21089 and ARG21060) were purchased from Arigo biolaboratories Crop. (Hsinchu, Taiwan). The primary antibodies (anti-PDL ab80276, anti-CD80 ab254579; anti-MHC CLASS II ab55152), rabbit monoclonal antibodies (anti-iba-1 ab108539, anti-CD86 ab53004), rabbit polyclonal antibodies (anti-IL1 beta ab9722) and secondary antibody (Goat Anti-Rat IgG H&L (FITC) and Goat Anti-Rabbit IgG H&L (Alexa Fluor 594, ab6840, ab150077 and ab150080), 3-(4,5-dimethylthiazol-2-yl)-5-(3-carboxymethoxyphenyl)-2-(4-sulfophenyl)-2 H-tetrazolium (MTS, ab223881) were purchased from Abcam (Cambridge, UK). The primary antibodies (anti-CD38-FITC 102,705, anti-iNOS 102,705), primary rat monoclonal antibodies (anti-F4/80 b158345) were purchased from Biolegend (San Diego, CA, USA). Interferon-γ was purchased from R&D systems (IFN-γ, 485-MI, Minneapolis, MN, USA). The TNF-*α* ELISA kit was purchased from R&D systems (DY410-05, Minneapolis, MN, USA). All chemicals were used directly without any further purification. Cucumarioside А_2_-2 was isolated from the sea cucumber *Cucumaria japonica* by standard procedures [25] and kindly provided by Drs Avilov S. A. and Silchenko A. S. (PIBOC FEBRAS, Vladivostok, Russia). The glycoside was individual by ^1^ H and ^13^С NMR.

### Ehrlich carcinoma cells

The museum tetraploid strain of mouse Ehrlich ascites carcinoma (EAC) was provided by the N.N. Blokhin Russian Oncology Center (Moscow, Russia). EAC cells were injected into the peritoneal cavity of Balb/c mice. Cells for experiments were collected 7 days after inoculation. For this purpose, mice were killed by cervical dislocation, and the ascitic fluid containing tumor cells was collected with a syringe. The cells were washed two times by centrifugation at 2000 rpm (450×g) for 10 min in PBS (pH 7.4) followed by resuspension in RPMI-1640 culture medium without serum. The cell number and viability were determined with a hemocytometer and the trypan blue staining procedure. The final cell concentration in the media was usually 1.5 × 10^4^ cells/mL.

### 4T1, Hs578T and MDA-MB-231 breast cancer cells

The human Hs578T cells and MDA-MB-231 cells were purchased from Bioresource Collection and Research Center (BCRC, # 60,001, # 60,120 and # 60,425), Taiwan. The mouse breast cancer cell line 4T1 cells were kindly provided by Dr. Shyng-Shiou F. Yuan (Kaohsiung Medical University, Kaohsiung City, Taiwan). RAW 264.7 cells, Hs578T cells and MDA-MB-231 cells were culture in DMEM with 10% heat-inactivated fetal bovine serum, 100 U/mL penicillin and 100 µg/mL streptomycin. The 4T1 cells were cultivated in DMEM with 10% heat-inactivated fetal bovine serum, 100 U/mL penicillin and 100 µg/mL streptomycin, sodium pyruvate, and non-essential amino acid. Cells were maintained in humidified atmosphere at 37 °C with 5% CO_2_.

### Preparation of activated peritoneal macrophages

Peritoneal macrophages were isolated from mouse peritoneal cavity [37]. After isolation, peritoneal macrophages were seeded in a 10 cm petri dish for 2 h. Then the peritoneal macrophages were treated with LPS (10 ng/mL) and IFN-γ (20 ng/mL), or LPS (10 ng/mL) and IFN-γ (20 ng/mL) combination with CA_2_-2 (0.01 µM), or CA_2_-2 (0.01 µM) alone for 2 or 24 h. Polarized macrophages were collected in microcentrifuge tubes (2.5 × 10^5^ cells/ml).

### Polarization of RAW 264.7 macrophages

RAW 264.7 cells were seeded into 6-well culture plate at a density of 3 × 10^5^ cells per well. After overnight incubation, CA_2_-2 (0.05 µM or 0.01 µM), or LPS (10 ng/mL) paired with IFN-γ (20 ng/mL), or CA_2_-2 (0.05 µM or 0.01 µM) paired with LPS (10 ng/mL) and IFN-γ (20 ng/mL) were added to each well for 24 h, in order to simultaneously induce M1 macrophages polarization [38].

### TNF-α secretion determination

TNF-α levels in culture media were determined by R&D Mouse TNF-α ELISA kit according to the manufacturer’s instruction. In brief, the 96-well plates were pre-coated with the capturing antibody at room temperature overnight. The cultured media of the polarized RAW 264.7 cells was collected and added to the plates at room temperature for 2 h. After three times washing, a biotinylated detection antibody was applied to the well at room temperature for 2 h. Plates were washed three times by wash buffer, and streptavidin-labeled HRP was added to the plates at room temperature for 20 min. The plates were further washed for three times. The levels of TNF-α were visualized by TMB substrate, and OD 450 was measured by a microplate reader.

### Measurement of ROS Content

Murine macrophages were plated into 96-well microplates and incubated at 37 ◦C with 5% CO_2_ for 2 h. After adhesion, cells were washed 3× and incubated with test compound (CA_2_-2: 1, 0.1, 0.01 µM and 0.001 µM) for 24 h (DMEM). For endogenously generated ROS detection studies, the cells were co-incubated with 2,7-dichlorodihydrorofluorescein diacetate (H2DCF-DA) solution (Molecular Probes, final concentration 10 µM) was added to each well, and the microplate was incubated for an additional 30 min at 37 °C. Prior to fluorescence registration, the cells were washed three times with PBS and then bathed in 200 µL/well of PBS. Green fluorescence of cells was registered at λex = 480 nm and λem = 520 nm. In each experiment LPS from *E. coli* serotype 055:B5 (1.0 µg/mL, Sigma-Aldrich, St. Louis, MO, USA) were used as a positive control. Fluorescent intensity was measured using plate reader PHERAstar FS (BMG Labtech GmbH, Ortenberg, Germany).

### Measurement of Nitric Oxide Content

For endogenously generated nitric oxide detection studies, peritoneal murine macrophages were incubated with CA_2_-2 (1, 0.1, 0.01 µM and 0.001 µM) for 24 h, then co-incubated with 10 µM of FA-OMe fluorescent probe for 8 h. Prior to fluorescence registration, the cells were washed three times with PBS and then bathed in 200 µL/well of PBS. Green fluorescence of cells was registered at λex = 480 nm and λem = 520 nm. In each experiment LPS from *E. coli* serotype 055:B5 (1.0 µg/mL, Sigma-Aldrich, St. Louis, MO, USA) were used as a positive control. Fluorescent intensity was measured using plate reader PHERAstar FS (BMG Labtech GmbH, Ortenberg, Germany).

### iNOS expression measurement

The Balb/c murine macrophages were incubated with CA_2_-2 (0.01 µM) or untreated for 15, 30, 60 and 240 min. Then cells were washed with PBS and total RNA was isolated from 1 × 10^6^ macrophages of each group using the TRIZOL reagent according to the manufacturer’s instructions (GIBCOBRL, Gaithersburg, MD). RNA was reverse transcribed into complementary DNA using an MMLV RT kit (Evrogen, Russia). Murine iNOS and β-actin DNA was amplified using the specific primers: iNOS F 5’ - CAG CTG GGC TGT ACA AAC CTT − 3’ and iNOS R 5’ - CAT TGG AAG TGA AGC GTT TCG − 3’; β-actin F 5’ - AGA GGG AAA TCG TGC GTG AC -3’ and β-actin R 5’ - CAA TAG TGA TGA CCT GGC CGT − 3’. Amplification was performed using a hot start protocol with 45 cycles of the following sequential steps: 96 °C for 15 s, 60 °C for 50 s. PCR products were visualized on a 3% agarose gels using ethidium bromide staining and identified in the expected positions on gels accordingly of its sizes.

### CD marker flow cytometry detection

Peritoneal murine macrophages cultured in DMEM supplemented with 10% FBS, 1% sodium pyruvate and 1% MEM nonessential amino acids at 37 °C under a humidified 5% CO_2_ atmosphere were co-incubated with different concentrations of CA_2_-2 (10 nM) and then subsequently by incubating with LPS (1 µg/mL) and IFN-γ (20 ng/mL) for 24 h at 37 °C for observing the change in M1 polarization. Polarized macrophages were collected in microcentrifuge tubes (1 × 10^6^ cells/tube), washed three times with PBS and then resuspended in 1 mL PBS in FACS tubes. Finally, the immunofluorescence of peritoneal murine macrophages was identified by anti-CD86, anti-CD38- FITC antibodies, anti-CD80, anti-MHC CLASS II, Goat Anti-Rabbit IgG Alexa Fluor 594 with the FACScalibur flow cytometer (Becton-Dickinson, Franklin Lakes, NJ, USA). Immunofluorescence of RAW 264.7 macrophages were identified by anti-mouse CD86 antibody conjugated with FITC and mouse MHC class II antibody conjugated with R-Phycoerythrin (RPE/Cy5). The fluorescence signals were detected by BD Accuri™ C6 flow cytometer (Becton-Dickinson, Franklin Lakes, NJ, USA).

### Macrophage cytotoxicity assay

Macrophages were co-incubated with target cells (EAC) for 24 or 48 h at 37 °C; their cytotoxic activity was then evaluated by measuring the residual target cell viability. The MTS colorimetric method was used to quantify this parameter. For M1 polarization, macrophages treated with LPS (100 ng/mL) + IFN-γ (40 ng/mL) or CA_2_-2 (0.05 µM) for 24 h. Cell density of macrophage was 3.6 × 10^4^ cells/well. Cell density of EAC cell was 3 × 10^3^ cells/well. Then cells were co-incubated in 96-well plates and cell viability was evaluated using MTS assay method. For this purpose, cells were incubated with 10 µL MTS reagent for 3 h, and the absorbance in each well was measured at 490/630 nm using a microplate reader PHERAstar FS (BMG Labtech, Germany). All the experiments were made in triplicate. Data were expressed as mean absorbance value (OD) of triplicate samples + standard error of the mean. Percentage-specific cytotoxicity (% C) was calculated as follows:

$$\left( {\% {\text{C}}} \right) = \frac{{({\text{OD}}\,{\text{macrophages}} + {\text{cancer}}\,{\text{cells}})}}{{\left( {{\text{OD}}\,{\text{macrophages}}} \right) + \left( {{\text{OD}}\,{\text{cancer}}\,{\text{cells}}} \right)}} \times 100$$

In case of determination of macrophage tumoricidal activity via paracrine manner, Hs578T, 4T1, and MDA-MB-231 cells were seeding at the density of 1.5 × 10^4^ in 96-well plates. After 24 h the cells were incubated with the conditioned-medium from polarized RAW 264.7 or M0 macrophages for 24 or 72 h. Then cell viability was evaluated using the 3-(4,5-dimethylthiazol-2-yl)-2,5-diphenyltetrazolium bromide (MTT) assay kit according to the manufacturer’s protocols. For this purpose, the culture medium was replaced with MTT-containing medium (0.25 mg/mL) and incubated for 4 h. The purple formazan crystals were re-suspended in DMSO and the absorbance at 540 nm was measured using an ELISA reader. All the experiments were repeated three times, and the mean absorbance values were calculated. The results were presented as percent of control data.

### Synthesis of fluorescent labeled nanoparticles

Preparation of nanoparticles was performed according to the method [39] with modifications. To synthesize silane-NH_2_-mPEG, N-Boc-ethylenediamine was conjugated to methoxypoly (ethylene glycol) acrylate (mPEG-Ac) by the Michael reaction, following the hydrolysis of Boc to (3-aminopropyl) triethoxy silane acrylate (APTES-Ac). A nanoparticle conjugate (MnMEIO-mPEG NPs) has been developed to prevent the non-specific binding of nanoparticles with cell membranes. The manganese-doped magnetic iron oxide (MnMEIO) NPs, composed of Fe/Mn with around 2.2 ratios, were synthesized by a non-hydrolytic thermolysis route. Silane-NH_2_-mPEG coated NPs (MnMEIO-mPEG NPs) were synthesized using oleic acid, oleyl amine-stabilized MnMEIO NPs, and silane-NH_2_-mPEG through a ligand-exchange reaction. The surface characteristic of NPs becomes hydrophilic after the ligand-exchange reaction. The carboxylic acid of CyTE777 dye was activated by EDC/NHS, which could conjugate with MnMEIO-mPEG NPs to form MnMEIO-CyTE777-mPEG NPs (NIR-NPs).

### Prussian blue staining assay

RAW264.7 cells were seeded at a density of 1 × 10^5^ cells/well on cover glasses (24 × 24 mm) and allowed to grow for 24 h. To determine whether the MnMEIO-mPEG-CyTE777 can be effectively engulfed by RAW 264.7 cells, RAW 264.7 cells were incubated with MnMEIO-mPEG-CyTE777 (0.25 µM) for 1, 2, 3, and 4 h, respectively. To detect Fe deposition, slides were stained with Prussian blue. First, cells and MnMEIO-mPEG-CyTE777 were incubated with 20% Hydrochloric acid(aq) at 55 °C for dissociated Fe. Second, the slide was stained with a 1:1 mixture of 10% potassium hexacyanoferrate (II) trihydrate and APS for 30 min at room temperature. The slides were rinsed with PBS buffer, dehydrated, and photographed.

### Animal model

BALB/c mice, weighing 18–20 g, were purchased from the Russian National Center for Genetic Resources of Laboratory Animals on the basis of the SPF vivarium (ICiG SB RAS, Novosibirsk, Russia). All mice were raised in an environmental with a 12-h light/dark cycle at a temperature 22 ± 1 °C with available food and water. To obtain ascite form of Ehrlich carcinoma the tumor cells were inoculated *i.p.* into the experimental animals (2.0 × 10^6^ cells in 0.5 mL of saline). To obtain solid version of Ehrlich carcinoma the tumor cells were hypodermically inoculated under the left shoulder blade of the mouse, 5 × 10^6^ cells/mouse in 0.5 mL of saline.

BALB/cAnN.Cg-*Foxn1*^nu^/CrlNarl mice (6 − 8 weeks old, female) were purchased from the National Laboratory Animal Center, Taipei, Taiwan. Animal experiments were performed following the institutional guidelines. 4T1-tumor cells in 50 µL PBS (1 × 10^6^ cells) and 50 µL matrigel were subcutaneously injected into the right flank of mice.

### In vivo 4T1 tumor growth and body weight changes

Nude mice (*n* = 3) bearing 4T1 tumors were subcutaneously injected near the tumor site with 5 × 10^5^ activated macrophages and non-activated macrophages (control) once every 3 days until day 15. The 4T1-bearing nude mice were sacrificed and the tumor growth of nude mice was measured on day 3, day 6, day 9, day 12 and day. Tumor size was measure by caliper measurement.

### Cellular anti-cancer immunotherapy in vivo against Ehrlich carcinoma

The effect of CA_2_-2 on the tumoricidal activity of peritoneal macrophages was investigated. The Ehrlich carcinoma tumors were maintained by intraperitoneal transplantation of 2.5 × 10^6^ cells/mouse. Mice were treated with untreated macrophages and macrophages treated with 0.02 µM CA_2_-2 ones a day in a volume of 0.5 mL during 3 days. Macrophages 1 × 10^6^ cells/mouse were injected into mice for 1, 3 and 7 days after inoculation of Ehrlich carcinoma. The control group of mice received the same volume of saline. Three groups of animals were formed with 8 mice in the group:

group 1 - control. The intact tumor cells were transplanted to animals. Then mice were treated with saline;

group 2 - untreated macrophages. The intact tumor cells were transplanted to animals. Then mice were injected with untreated macrophages;

group 3 - MO macrophages treated with 0.02 µM CA_2_-2. The intact tumor cells were transplanted to animals. Then mice were injected with macrophages treated with 0.02 µM CA_2_-2.

Observation of the animals was continued for 42 days. The average life span (ALS) for mice bearing tumors was determined for all groups of animals. The increase in life span (ILS; %) was determined according to the following equation: ILS = (T/C) × 100%, where T and C are the ALS (days) of animals in the experimental and the control groups.

### In vivo optical imaging study

Peritoneal macrophages were seeded in a 6-well plate with a density of 2.5 × 10^6^ cells/well. For macrophage activation, the peritoneal macrophages were treated with LPS (10 ng/mL) and IFN-γ (20 ng/mL), or LPS (10 ng/mL) and IFN-γ (20 ng/mL) combination with CA_2_-2 (0.01 µM), or CA_2_-2 (0.01 µM) alone for 24 h. Then the activated macrophages were incorporated with MnMEIO-mPEG-CyTE777. Nude mice bearing 4T1 tumors (n = 3) were anesthetized with an inspired concentration of 1.5% isoflurane with 2 L/min of oxygen and subcutaneously injected with MnMEIO-mPEG-CyTE777-target macrophages. Optical imaging was acquired at pre-injection and different time points (2, 4, 6, 8, and 24 h) after injection using an IVIS spectrum system (PerkinElmer, Waltham, MA, USA). Then the emission at 820 nm was measured with an optimal excitation wavelength of 745 nm.

### Statistical analysis

Experiments were repeated at least three times. Statistical analysis was performed using GraphPad Prism 7.0 software (GraphPad Software Inc., CA). One-way and two-way analyses of variance (ANOVA) with a Tukey post-hoc test was used for multiple comparisons, and Student’s *t*-test was used for two-group comparisons. All results are presented as means ± SEM, and differences with *p* < 0.05 were considered significant.

## Results

### CA_2_-2 induces polarization of M0 macrophages to M1 macrophages through increased TNF-α secretion, ROS and NO production, iNOS expression, and upregulation of CD markers

**Fig. 2:**
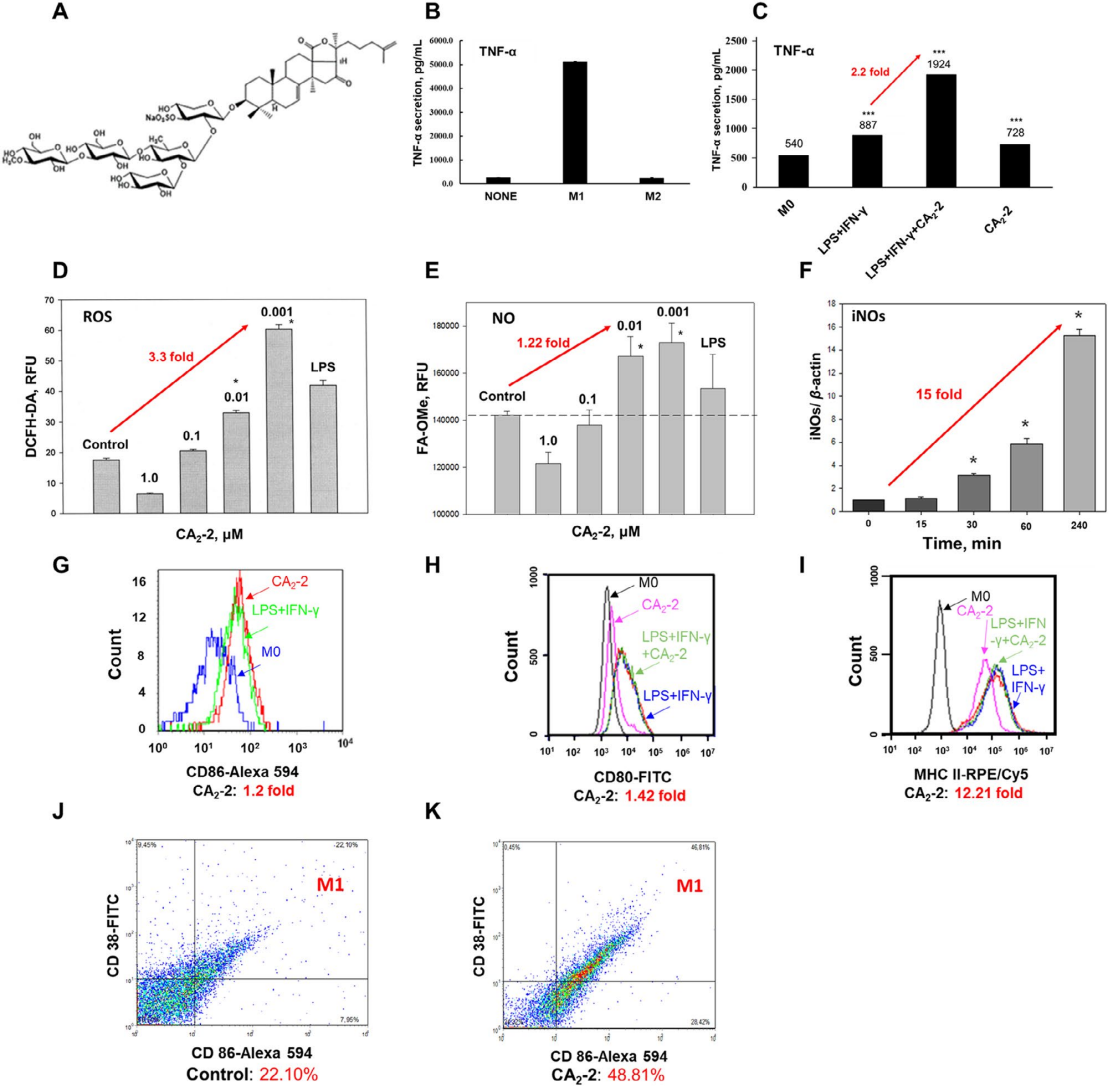
CA_2_-2 increases the level of TNF-a, ROS, NO, iNOS, and some CD markers in macrophages. **(A)** chemical structure of CA_2_-2. **(B)** ELISA analysis of TNF-α secretion by macrophage RAW 264.7 cells. The culture supernatants were harvested after treatment with LPS (1 µg/mL) and IFN-γ (20 ng/mL) for M1 polarization; IL-4 (20 ng/mL) for M2 polarization, and none treatment (M0) for 24 h. **(C)** TNF-α secretion by macrophage RAW 264.7 cells. The culture supernatants were harvested after cell co-treatment with LPS (1 µg/mL) and IFN-γ (20 ng/mL) and CA_2_-2 (0.01 µM) for 24 h. **(D)** Effect of CA_2_-2 on the ROS content in mouse peritoneal macrophages. The incubation time with cells is 2 h. **(E)** Effect of CA_2_-2 on the NO content in RAW 264.7 cells. The incubation time with cells is 2 h. The concentration of LPS used as positive control is 0.5 µg/ml. **(F)** Effect of CA_2_-2 on the level of iNOS expression in peritoneal macrophages. The level of iNOS expression is normalized to the level of beta-actin expression. The concentration of CA_2_-2 is 0.01 µM, incubation time of cells with the glycoside: 0 min (control), 15 min, 30 min, 1 h, and 4 h. **(G-I)** CA_2_-2 increases CD86, CD80, and MHC CLASS II markers in RAW 264.7 macrophages. The meaning of different colors is: (**G**) blue – nonactivated macrophages M0; green – activated by LPS + IFN-γ macrophages M1; red – activated by CA_2_-2 macrophages M1; (**H-I**) black – nonactivated macrophages M0; pink – activated by CA_2_-2 macrophages M1; blue – activated by LPS (10 ng/mL) + IFN-γ macrophages M1; red – activated by LPS (1 µg/mL) + IFN-γ + CA_2_-2 macrophages M1; green – activated by activated by LPS + IFN-γ + CA_2_-2 macrophages. **(J-K)** CA_2_-2 increases CD86 and CD38 markers in mouse peritoneal macrophages. Data are presented as mean ± SE (*n* = 5). *p < 0.05 compared to control; *** *p* < 0.001 compared to M0 macrophages

In order to demonstrate that the triterpene glycoside CA_2_-2 is able to activate and polarize mouse macrophages into M1 phenotype, we studied the effect of this compound on the expression of a number of macrophage markers associated with the transition from M0 to M1. To this purpose, we evaluated the secretion of cytokines such as TNF-α, the production of reactive oxygen species, NO, the activity of the iNOS enzyme in different types of mouse macrophages, and the appearance of surface CD markers specific to the M1 macrophages. Incubation of macrophage RAW 264.7 cells in the presence of CA_2_-2 led to an increase in the production of tumor necrosis factor-alfa (TNF-α) (Fig. 2A). Macrophages stimulated with classical inducers of M1 polarization, LPS and IFN-γ (LPS + IFN-γ), produce a significantly larger amount of TNF-α compared with intact cells and macrophages stimulated with interleukin IL-4 for their polarization in the M2 phenotype (Fig. 2B). While CA_2_-2 (0.01 µM) was added to the culture medium during incubation of cells with LPS + IFN-γ inducers, TNF-α production was increased 2.2-fold compared with cells polarized in the M1 phenotype without glycoside. Moreover, macrophages incubated in the presence of CA_2_-2 alone led to a significant increase in TNF-α production in these cells (Fig. 2C). Thus, a low concentration of CA_2_-2 boosts TNF-α secretion in macrophages cultivated with LPS + IFN-γ or alone for M1 polarization. Furthermore, incubation of mouse peritoneal macrophages (Fig. 2D) or RAW 264.7 macrophages (data not shown) with glycoside in various concentrations led to a dose-dependent change in the ROS content. The most effective stimulating glycoside concentration was 0.001 µM, at which the ROS level in the cells increased by 3.3 times compared with untreated cells (Fig. 2D). However, CA_2_-2 at 0.1 µM was practically ineffective. Moreover, when CA_2_-2 was used at a subtoxic concentration of 1.0 µM, a significant inhibition of ROS production in cells was observed compared to the control level (Fig. 2D). The cytotoxicity (EC_50_) of CA_2_-2 for mouse macrophages is 3.0 µM [30].

Incubation of peritoneal macrophages with CA_2_-2 in nanomolar concentrations led to a significant increase in NO content in the cell cytoplasm. An inversely proportional concentration dependence of CA_2_-2 action on the NO level in the cells was observed. The highest stimulating effect was noted for glycoside concentrations of 0.001 and 0.01 µM, which raised NO content by approximately 1.2 times compared with control cells. At the same time, a 1.0 µM glycoside led to a pronounced inhibition of NO production (Fig. 2E). To rule out the source of the nitric oxide in the macrophage after CA_2_-2 administration, macrophages were incubated in the presence of CA_2_-2 for various periods of time. Then, an analysis of the iNOS expression by RT-PCR was performed. The result showed that CA_2_-2 in the concentration of 0.01 µM noticeably stimulated NO production and caused significant activation of iNOS gene expression in the macrophages. The maximum increase in the expression of the iNOS gene by 15-fold was observed 4 h after the incubation with CA_2_-2 (Fig. 2F).

To further substantiate the M1 polarization of macrophages after CA_2_-2 administration, several CD markers on the cell surface representing the M1 phenotype were evaluated. The cultivation of RAW 264.7 macrophage cells in the presence of CA_2_-2 (0.01 µM) led to an increase in CD80 by 1.4-fold, CD86 by 1.2-fold, and MHC class II by 12.2-fold (Fig. 1G-I). Under CA_2_-2 action, the expression of CD markers specific to M1 phenotype also increased in mouse peritoneal macrophages. The number of cells with detectable CD38 and CD86 doubled compared to intact macrophages (Fig. 2J-K).

Collectively, we found that the triterpene glycoside from the holothurian, CA_2_-2, is able to activate and polarize different mouse macrophages into the M1 phenotype at nanomolar concentrations in the range of 1.0–50.0 nM.

### CA_2_-2 increases the tumoricidal activity of RAW 264.7 and mouse peritoneal macrophages against cancer cells in a paracrine manner in vitro

**Fig. 3:**
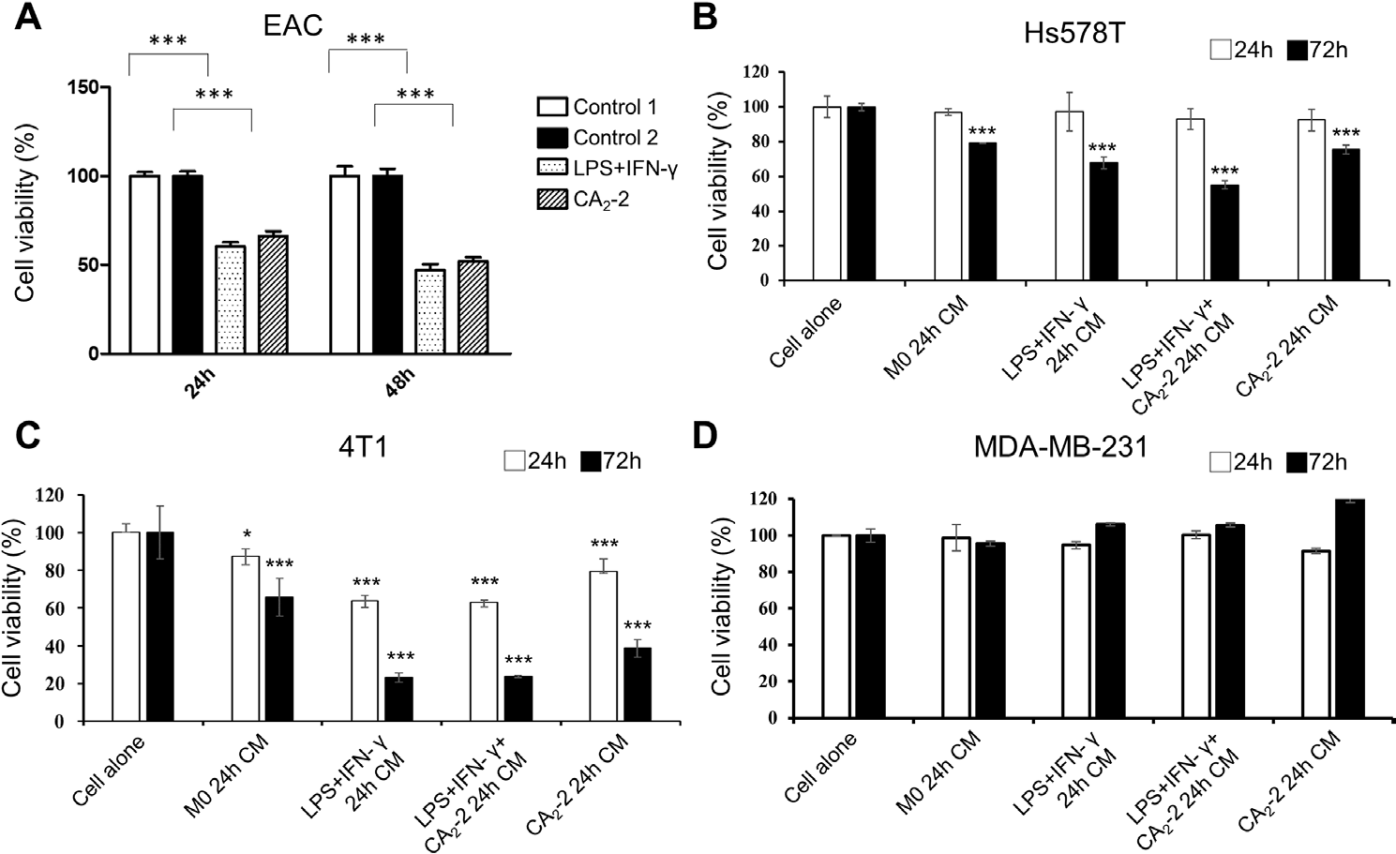
Cytotoxic activity of mouse macrophages against cancer cells. **(A)** Mouse peritoneal macrophages were polarized to M1 phenotype by preincubation with LPS (100 ng/mL) + IFN-γ (40 ng/mL) or CA_2_-2 (0.05 µM) alone. EAC cells were incubated with polarized macrophages for 24 or 48 h. Macrophage density was 3.6 × 10^4^ cells/well; EAC cell density was 3 × 10^3^ cells/well. Cell viability was detected by the MTS method. **(B-D)** The cytotoxic activity of RAW 264.7 macrophages against different triple-negative breast cancer cells via paracrine manner. Culture medium (CM) was obtained from RAW 264.7 macrophages treated with CA_2_-2 (0.05 µM) alone or with CA_2_-2 (0.05 µM) and LPS (10 ng/mL) + IFN-γ (20 ng/mL) for M1 polarization for 24 h. Control macrophages were treated with DMEM medium only for M0 phase. Cancer cells were incubated with CM for 24 and 72 h. Data are expressed as the means ± SEM (*n* = 5). *p < 0.05, ***p < 0.001 compared to control

Next, we aim to determine whether the cytotoxic activity of M1 macrophages toward tumor cells was enhanced. To this end, we investigated the ability of both mouse macrophages cell line and primary cells treated with CA_2_-2 to suppress the viability of several tumor cell lines, including mouse Ehrlich ascites carcinoma (EAC) cells, mouse 4T1 triple-negative breast cancer cells, human Hs578T, and MDA-MB-231 triple-negative breast cancer cell lines. Incubation of macrophages with LPS + IFN-γ or CA_2_-2 and subsequent co-incubation of M1 macrophages with mouse EAC cells in a 10:1 ratio (effector: target) resulted in the decrease of cancer cell viability in vitro (Fig. 3A). Polarization of macrophages with CA_2_-2 resulted in approximately 40–50% loss of tumor cell viability. Similar cytotoxic activity was also exhibited by macrophages previously polarized by standard LPS + IFN-γ inducers. Prolonged incubation time of macrophages with tumor cells from 24 to 48 h led to a more significant decrease in the EAC cell viability.

Mouse macrophage cells RAW 264.7 activated and polarized to M1 phenotype by CA_2_-2 were found to exhibit significant cytotoxic activity against triple-negative breast cancer (TNBC) cells via paracrine manner. The culture medium of RAW 264.7 macrophages polarized to M1 phenotype by cell incubation with LPS + IFN-γ + CA_2_-2 or CA_2_-2 alone reduced mouse 4T1 cancer cell viability by 75% and 60%, respectively. They also decreased human Hs578T cancer cell viability by around 45% and 25%, respectively. The most pronounced cytotoxic activity was detected after 72 h of incubation. No significant anticancer activity was observed in human MDA-MB-231 cells (Fig. 3B-D). Taken together, these data suggested that CA_2_-2-treated macrophages could not only reduce the viability of mouse tumor cells (EAC and 4T1 cell lines) but also some human tumor cells (Hs578T).

### Synthesis of CyTE777-labeled nanoparticles

**Fig. 4:**
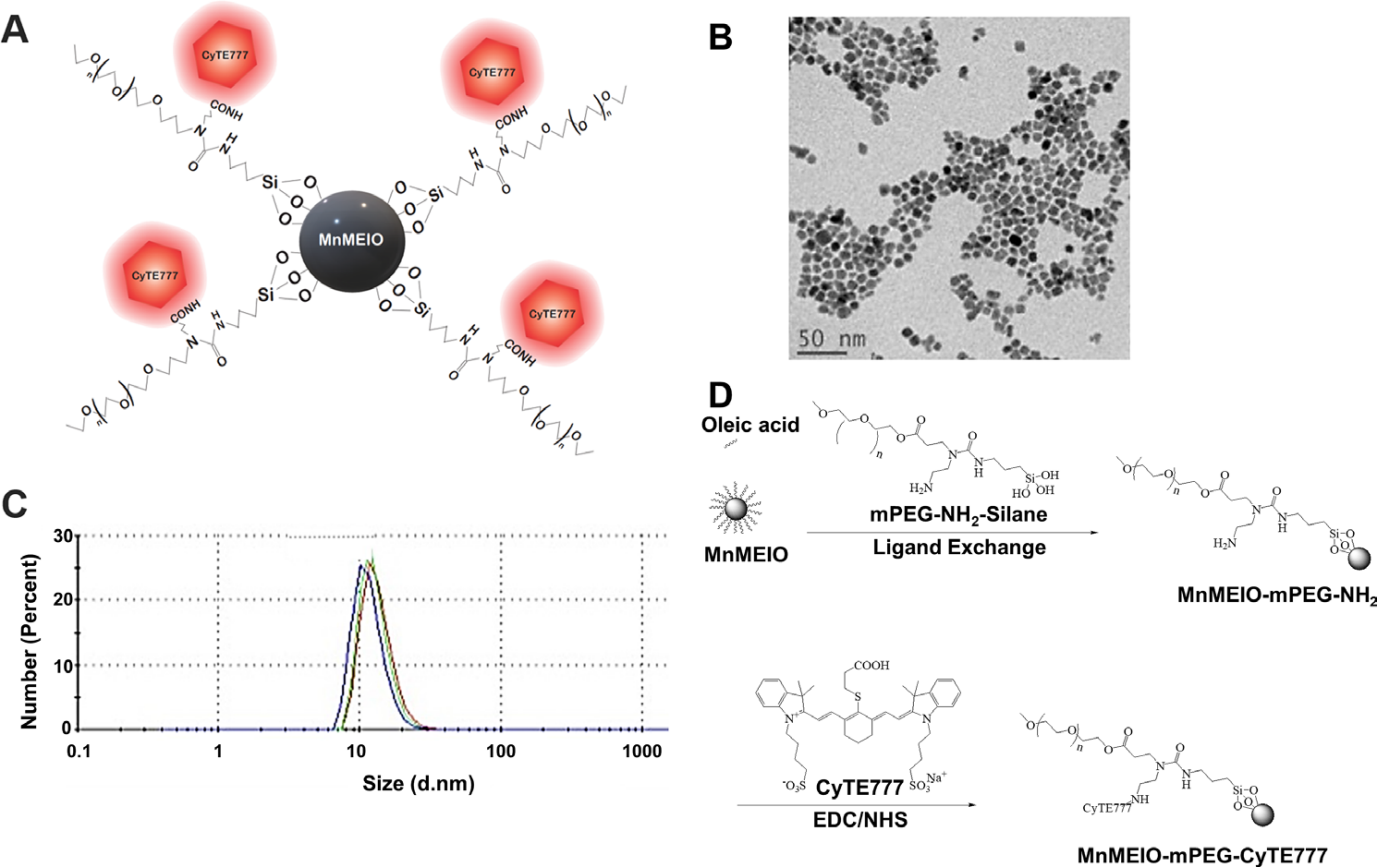
Fluorescent labeled MnMEIO-mPEG-CyTE777 nanoparticles (NIR-NPs) development [39]. **(A)** Schematic presentation showing the structure of MnMEIO-mPEG-CyTE777 nanoparticles. **(B)** TEM image showing the spherical and monodispersed morphology of NIR-NPs. Scale bar, 50 nm. **(C)** Number-average diameters of NIR-NPs as determined by dynamic light scattering (DLS). **(D)** Synthesis of MnMEIO-mPEG nanoparticles and introduction of a near-infrared fluorescent probe, CyTE777, into the structure of MnMEIO-mPEG nanoparticles

To monitor M1 macrophage targeting in tumor-bearing mice, we synthesized nanoparticles labeled with near-infrared fluorochrome and optimized the conditions for M1 macrophage loading with these nanoparticles. The manganese-doped iron oxide core (MnMEIO) nanoparticles were obtained by a non-hydrolytic thermal decomposition process. Nanoparticles conjugated with a near-infrared fluorochrome were synthesized specifically to track macrophages in the body of tumor-bearing animals with optical imaging (Fig. 4A-D). The NIR-NPs were composed of a MnMEIO, a silane-amino functionalized poly(ethylene glycol) copolymer shell, and a near-infrared fluorescence dye (CyTE777) (Fig. 4A). The low autofluorescence and deep tissue penetration properties of CyTE777 enable its application for in vitro and in vivo optical imaging studies. Transmission electron microscopy (TEM) image of NIR-NPs demonstrated the formation of uniformly spherical nanoparticles (Fig. 4B). The spherical nanoparticles possess a hydrodynamic size of 19.81 ± 0.20 nm (Fig. 4C), zeta potential values of 24.73 ± 3.22 Mv, and a polydispersity index (PDI) of 0.039.

### CA_2_-2-treated M1 macrophages efficiently uptake and label with NIR-NPs

**Fig. 5:**
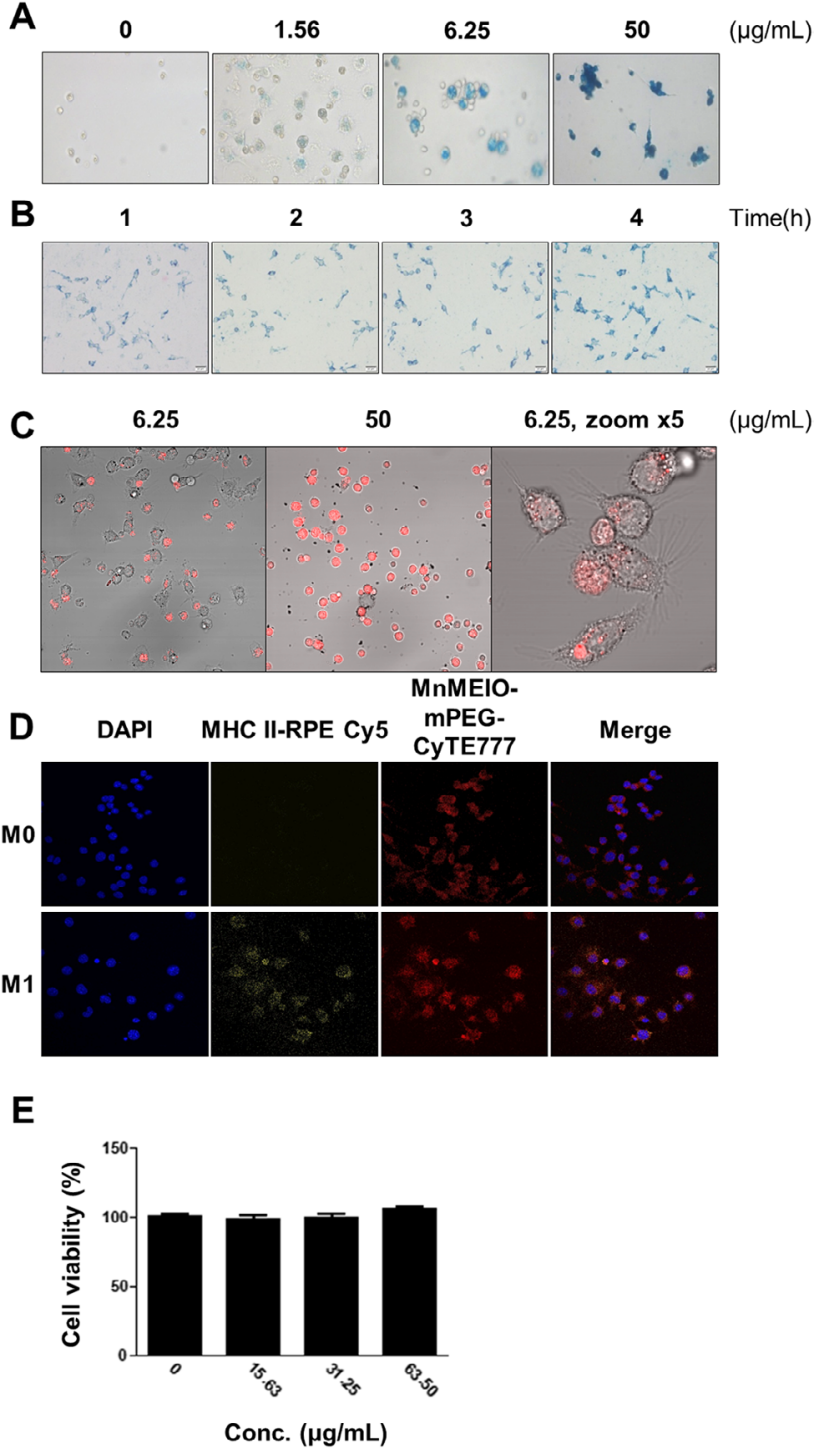
Macrophage loading with MnMEIO-mPEG nanoparticles. **(A)** Mouse peritoneal macrophages loaded with NIR-NPs for 1 h at different concentrations and stained with Prussian blue. **(B)** RAW 264.7 cells loaded with 0.5 µg/ml NIR-NPs at different time points (1 h, 2 h, 3 h, and 4 h). **(C)** Mouse peritoneal macrophages loaded with NIR-NPs at different concentrations, confocal microscopy. **(D)** RAW 264.7 cells treated with LPS + IFN-γ + CA_2_-2 for M1 polarization, and co-cultured with NIR-NPs for 1 h. The cell was stained with DAPI (nucleus, blue color), anti-MHC CLASS II-RPE/Cy5 (M1 marker, biomembrane, green color), and visualized with confocal microscopy. **(E)** The cytotoxicity effect of NIR-NPs in RAW 264.7 macrophages. The NIR-NPs were incubated with RAW264.7 macrophages at different dose for 24 h. Data are expressed as the means ± SEM (*n* = 5)

Various loading conditions of mouse peritoneal macrophages with MnMEIO-mPEG nanoparticles (NPs) were studied (Fig. 5A, B). At a density of peritoneal macrophages of 1–2 million/ml and a loading time of 1 h, the NPs are optimally loaded into cells when used in the concentration range of 1.56–6.25 µg/ml (Fig. 5A). Similar results were obtained with RAW 264.7 cell line: NPs at a concentration of 0.5 µg/ml, capable of effective loading within 1 h (Fig. 5B). Using NPs staining in cells with Prussian blue dye, mouse peritoneal macrophages and RAW 264.7 cells stably held nanoparticles for several hours to several days (Fig. 5A and B), and nanoparticles did not exhibit cytotoxic properties to macrophages detected using MTS method (Fig. 5E).

Fluorescence images of cells obtained by confocal microscopy showed that clusters of NPs conjugated with a near-infrared fluorescent probe, CyTE777 (MnMEIO-mPEG-CyTE777, NIR-NPs), were located in the cytoplasm of macrophage cells, and not on the nuclei or the outer surface of the cytoplasmic membrane (Fig. 5C and D). Thus, loading newly synthesized NIR-NPs into macrophages made it possible to track CA_2_-2-treated M1 macrophages and locate them in tumors in vivo.

### In vitro polarization of macrophages by CA_2_-2 sustained in tumor tissue in vivo

**Fig. 6:**
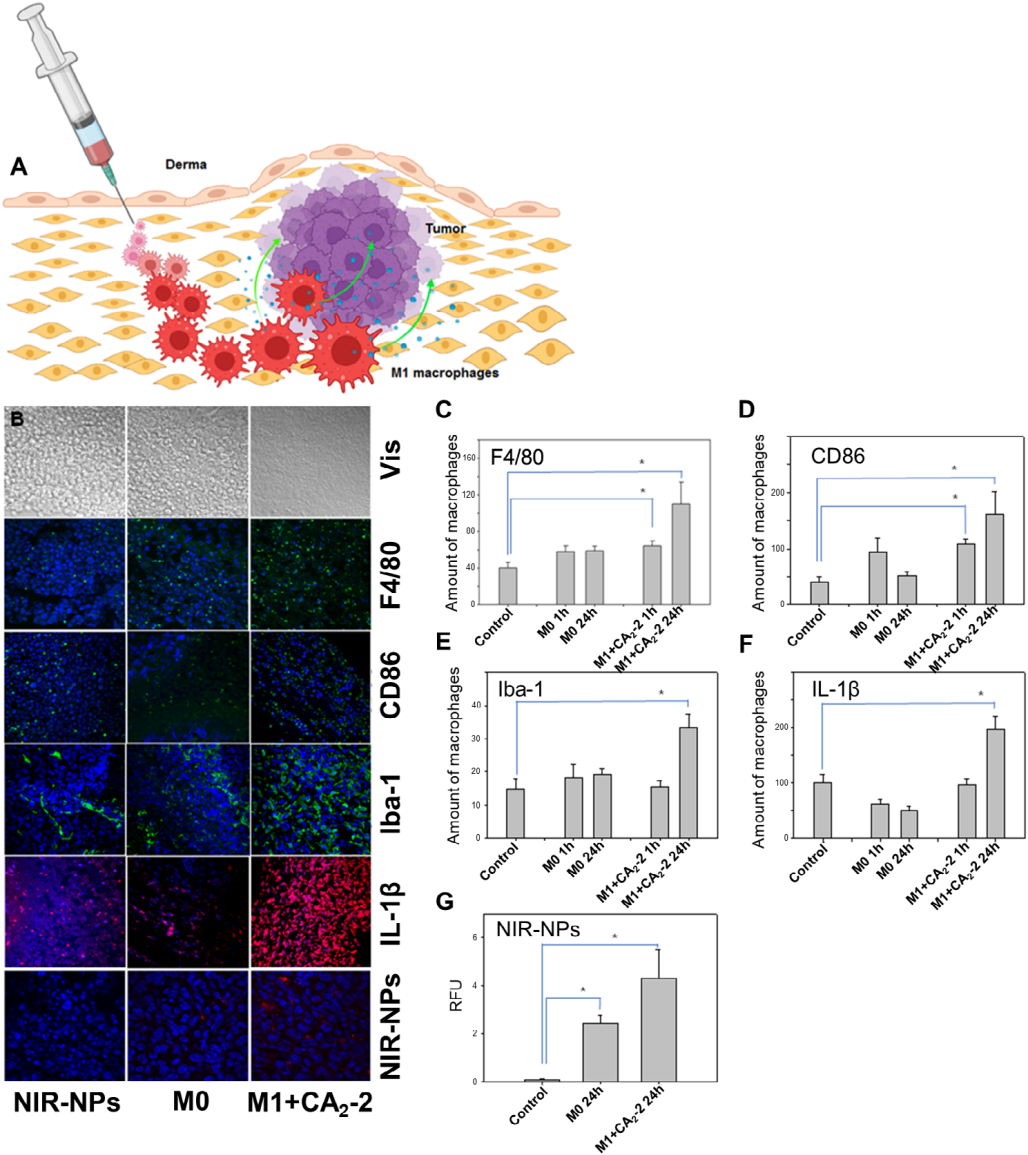
CA_2_-2 increases the amount of activated M1 macrophages inside tumor tissue after macrophage injection in the near tumor region. **(A)** General scheme of macrophage administration. **(B)** Immunohistochemical detection of BALB/c mouse peritoneal macrophages in cryosections of mouse solid Ehrlich carcinoma tissue 24 h after subcutaneous injection of macrophages at a concentration of 1 × 10^5^ cells/mouse. Macrophages were stained with antibodies to the F4/80 marker conjugated with FITC (green fluorescence), to CD86 marker conjugated with FITC (green fluorescence), to Iba-1 marker conjugated with FITC (green fluorescence), to IL-1β cytokine conjugated with TRITC (red fluorescence); NIR-NPs have a red fluorescence; the nuclei were stained with Hoechst 33,342 (blue fluorescence). The figure shows sections of a tumor from 3 groups of mice: the control group, the group receiving non-activated M0 peritoneal macrophages and the group receiving CA_2_-2 pre-treated (0.02 µM, 2 h) M1 peritoneal macrophages. Quantitative analysis of the content of peritoneal macrophages in tumor tissue. **(C)** Amount of F4/80 + cells. **(D)** Amount of CD86 + cells. **(E)** Amount of Iba-1 + cells; **(F)** amount of IL-1β producing cells; and **(G)** number of macrophages loaded with NIR-NPs in cryosections of Ehrlich carcinoma tissue 1 and 24 h after subcutaneous injection of macrophages. Groups of mice: control group; a group that received non-treated M0 peritoneal macrophages (1 and 24 h after the injection); the group that received CA_2_-2 pre-treated (0.02 µM, 2 h) M1 peritoneal macrophages (1 and 24 h after injection). Fluorescent images and images in transmitted light were obtained using a LSM510 META confocal microscope (Carl Zeiss, Germany). Image processing was carried out using the software ZEN 2012 lite (Carl Zeiss, Germany). Data are presented as mean ± SE (*n* = 8). *p < 0.05 compared to control

To carry out cell antitumor therapy, we used mouse peritoneal macrophages previously in vitro activated and polarized into M1 phenotype by cell incubating with a nanomolar concentration of CA_2_-2. Then, the mice having a formed Ehrlich carcinoma were subcutaneously injected with NIR-NPs-targeted macrophages adjacent to the tumor sites (Fig. 6A). The number of polarized M1 macrophages that appeared inside the tumor tissues was counted at the indicated time. The immunohistochemical study of tumor tissue sections showed that the number of activated and polarized M1 macrophages in the tumor tissue significantly increased. F4/80 + macrophages with increased M1 marker CD86, activation marker iba-1 expression, and pro-inflammatory cytokine IL-1β production were detected in tumor tissue. Moreover, the most significant differences in the quantitative content of activated M1 macrophages were observed 24 h after the administration of the polarized M1 macrophages, whereas the number of activated M1 macrophages in the tumor was significantly lower 1 h after administration. Subcutaneous administration of non-activated and non-polarized macrophages (M0 phenotype) to mice did not increase activated M1 macrophages in Ehrlich carcinoma tissues (Fig. 6B-F).

The creation of NIR-NPs and their subsequent loading into peritoneal macrophages allowed us to directly track the appearance of these cells in the tumor tissues after their administration to experimental animals. The confocal microscopy imaging of tumor tissue sections showed that CA_2_-2-induced M1 macrophages penetrated faster than M0 macrophages into Ehrlich carcinoma tissues (Fig. 6B and G). Thus, activated and polarized M1 macrophages accumulated in the tumor tissue 24 h after the introduction to tumor-bearing animals. M1 macrophages in Ehrlich carcinoma were 2-fold higher than the control mouse tumor tissues or mice injected with inactive and non-polarized M0 macrophages. These data suggest that in vitro polarized M1 macrophages by CA_2_-2 could penetrate and sustain the M1 phenotype in tumor tissue in vivo.

### CA_2_-2-polarized М1 macrophages recognize the mouse 4T1 tumor in vivo

**Fig. 7:**
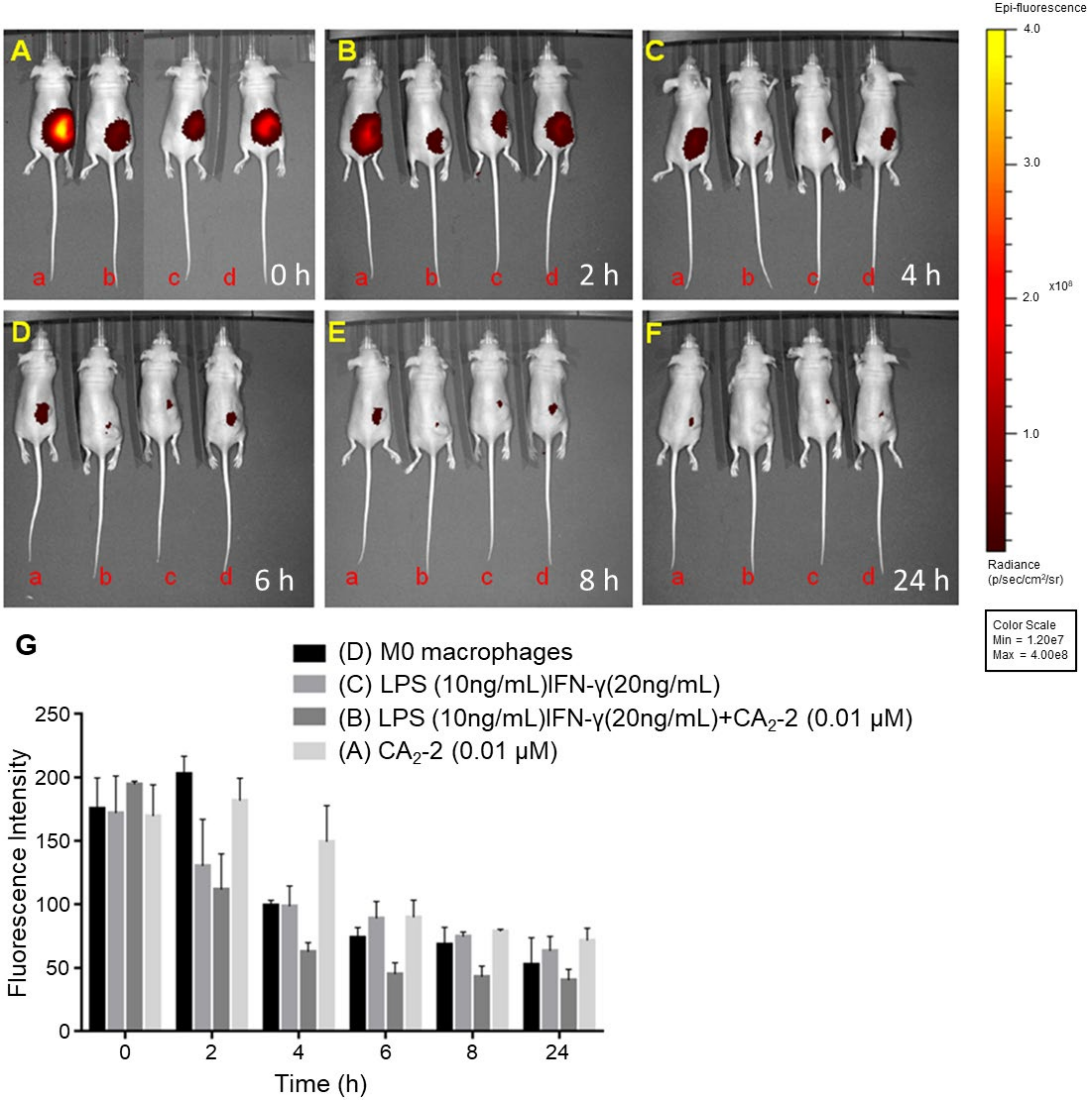
CA_2_-2 activated and polarized М1 macrophages recognize the mouse 4T1 tumor. Optical imaging of BALB/cAnN.Cg-*Foxn1*^nu^/CrlNarl 4T1 tumor-bearing mice infused by NIR-NPs targeted and CA_2_-2 stimulated macrophages near the tumor site at different time points (0, 2, 4, 6, 8, and 24 h). The figure shows the front view. Panels **(A), (B), (C), (D), (E)**, and **(F)** indicate the time points at 0, 2, 4, 6, 8, and 24 h, respectively. **a** – Represents mice injected with CA_2_-2 (0.01 µM)-stimulated macrophages. **b** – Represents mice injected with LPS (10 ng/mL) + IFN-γ (20 ng/mL) + CA_2_-2 (0.01 µM)-stimulated macrophages. **c** – Represents mice injected with LPS (10 ng/mL) + IFN-γ (20 ng/mL)-stimulated macrophages. **d** – Represents mice injected with M0 macrophages. Optical imaging was acquired using an IVIS spectrum system. Emission at 820 nm was measured with an optimal excitation wavelength of 745 nm. Panel **(G)** Quantification graphic of macrophage-NPs dynamics in tumor over time (h)

To investigate whether activated macrophages can target cancer cells in vivo, the M1 macrophages loaded with NIR-NPs were injected into tumor-bearing mice and monitored by IVIS imaging. For this purpose, the primary mouse peritoneal macrophages were activated by different stimuli such as LPS (10 ng/mL) + IFN-γ (20 ng/mL); LPS (10 ng/mL) + IFN-γ (20 ng/mL) + CA_2_-2 (0.01 µM), and CA_2_-2 (0.01 µM) alone for 24 h. After activation and polarization, the macrophages were incubated with NIR-NPs for 3 h to uptake the nanoparticles. Nude mice bearing subcutaneous 4T1 tumors were injected with NIR-NPs-targeted macrophages adjacent to tumor sites. The whole-body optical imaging was acquired by an IVIS system at different time points (0, 2, 4, 6, 8, and 24 h). The activated, polarized, and NIR-NPs-loaded macrophages reached the tumor sites immediately after injection, as shown in Fig. 7A, and then their number gradually decreased. However, the signals persisted for 24 h in the NIR-NPs-loaded macrophages (Fig. 7A-G). The fluorescent intensity in the 4T1-bearing nude mice injected with CA_2_-2-stimulated macrophages was slightly stronger than the groups injected with LPS + IFN-γ, LPS + IFN-γ + CA_2_-2-treated cells, or M0 macrophages within 24 h. The quantification graphic of macrophage-NPs dynamics in tumor over time is shown in Fig. 7G. Overall, CA_2_-2-activated macrophages can target the tumor sites for 24 h, and their amount may be higher than non-stimulated macrophages. Moreover, NIR-NPs labeling allows the real-time tracking of the macrophages in vivo.

### CA_2_-2-educated peritoneal M1 macrophages suppress cancer cell growth in the Ehrlich carcinoma and 4T1 syngeneic mouse models

**Fig. 8:**
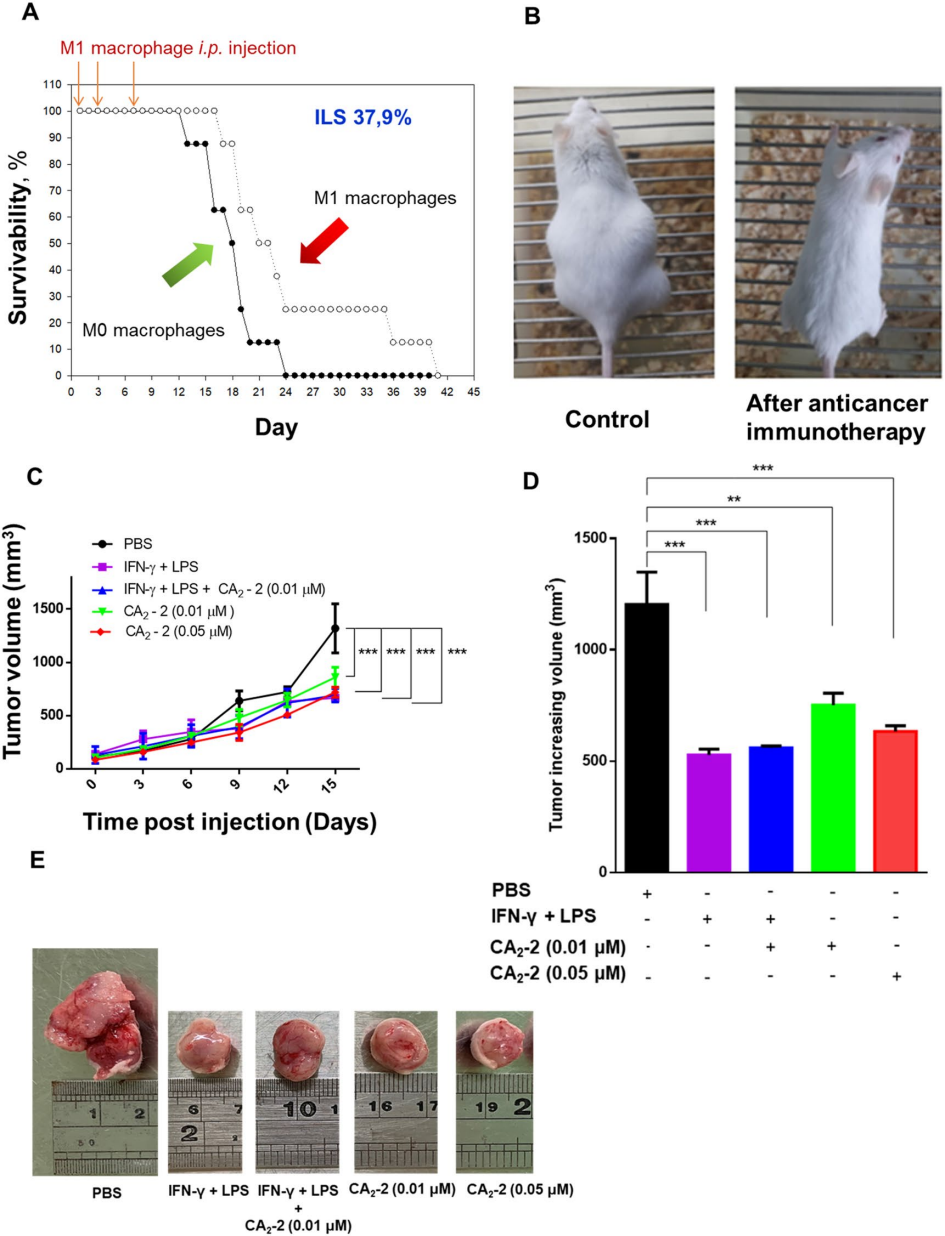
CA_2_-2-treated M1 macrophages exhibit antitumor activity. *in vivo.* **(A)** Survival of mice with Ehrlich ascites carcinoma (2 × 10^6^ cells/mouse) in groups receiving cell therapy with peritoneal macrophages and peritoneal macrophages pre-treated with CA_2_-2 (0.02 µM, 2 h) administered intraperitoneally at a concentration of 1 × 10^5^ cells/mouse at 1, 3, and 7 days after tumor inoculation. Increase in life span (ILS) of mice receiving M1 macrophages in expressed in %. **(B)** Representative image of mice with ascite Ehrlich carcinoma on 20 day of experiment in control group (left) and after injection with CA_2_-2-treated M1 macrophages. **(C)** Effect of activated primary macrophages on the nude mouse bearing 4T1 breast cancer. **(D)** Change in tumor size. The tumor increasing volume of different groups. **(E)** Representative image of mice with 4T1 tumor on 15 day of experiment in control group (left) and after injection with LPS (10 ng/mL) + IFN-γ (20 ng/mL), LPS (10 ng/mL) + IFN-γ (20 ng/mL) + CA_2_-2 (0.01 µM), and CA_2_-2 (0.01 µM or 0.05 µM)-treated macrophages. Data are presented as mean ± SE (*n* = 3). **p < 0.01, ***p < 0.001 compared to control

To explore the anti-cancer efficacy of the Ca2-2-polarized M1 macrophages in vivo, the ex vivo CA2-2-treated and polarized M1 macrophages were injected into Ehrlich carcinoma and 4T1 syngeneic mouse models. The antitumor effect of such cell therapy was evaluated by the number of surviving animals and the increase in the average life span of mice. Control animals with Ehrlich ascites carcinoma died by the 15th–16th day after inoculation of the tumors. The average life span of animals treated with non-activated (M0 phenotype) macrophage cell therapy was 18.1 days, while the average life span of animals that were injected with CA_2_-2 pre-treated macrophages (M1 phenotype) was 25.0 days, suggesting an increase in life span (ILS) by 37.9% compared with mice received non-treated macrophages (M0 phenotype) (Fig. 8A and B).

CA_2_-2-treated М1 macrophages attenuated tumor growth rate in 4T1-bearing nude mice and changed the ratio of M2/M1 macrophages in 4T1 tumor tissues. In this study, the anti-cancer effect of activated primary macrophages was determined. 4T1 tumor-bearing nude mice were *i.p.* injected with activated macrophages once every 3 days in the dose of 2 × 10^6^ cells/mouse. The results showed that the CA_2_-2, LPS + IFN-γ, and LPS + IFN-γ + CA_2_-2-activated macrophage administration significantly reduced tumor volume in the 4T1 bearing nude mice 15 days post-injection compared to the PBS-treated M0 macrophage, as shown in Fig. 8C and D. Tumor growth in mice treated with LPS + IFN-γ + CA_2_-2 and CA_2_-2 (0.05 µM)-activated macrophages were slower than the M0 macrophage (Fig. 8E), indicating M1 macrophages can suppress tumor growth. Together, these data suggest that CA_2_-2-educated M1 macrophages could suppress cancer growth and prolong mouse survival in the syngeneic mouse model.

## Discussion

In the present study, macrophage activation using LPS and IFN-γ as inductors was accompanied by increased expression of specific markers, CD38, CD80, CD86, and MHC CLASS II, increased secretion of pro-inflammatory cytokine TNF-α, the elevated production of ROS and NO, and accelerated expression of iNOS. CA_2_-2 alone or in combination with LPS + IFN-γ was shown to amplify these parameters, indicating the polarization toward the M1 phenotype. In some cases, a synergistic effect of the combined action of LPS + IFN-γ + CA_2_-2 was observed exceeding the effect of each of the effectors, probably due to its different molecular mechanisms of macrophage activation. Noteworthy, both peritoneal macrophages and RAW 264.7 cells were similarly activated and polarized into M1 phenotype with CA_2_-2, suggesting a generalizable mechanism of cell activation by glycoside.

We assumed that such polarization should increase the antitumor activity of macrophages. Experiments were conducted to study both the direct cytotoxicity of polarized M1 macrophages or via paracrine manner when cancer cells were incubated in conditional media where macrophages were polarized into M1 phenotype. Indeed, increased cytotoxicity of macrophages against mouse 4T1 TNBC cells, human Hs578T cells, and mouse EAC cells was found. The most sensitive cells to the macrophage cytotoxicity were the tumors originated from the mice (EAC and 4T1 cell), while human tumor cells (Hs578T cells and especially MDA-MB-231 cells) were significantly less susceptible or completely non-sensitive to the cytotoxicity of the macrophages. This may be due to the species-specificity of antitumor agents produced by the macrophages or to a different antitumor resistance characteristic of tumor cell lines. MDA-MB-231 cells are known as a very aggressive form of breast cancer, resistant to many cytostatics and with limited treatment options.

MnMEIO-mPEG-CyTE777 (NIR-NPs) is a multifunctional nanoparticle for optical and MR imaging developed by our lab [39]. In this study, NIR-NPs were incorporated into macrophages through phagocytosis. Glycoside-activated macrophages more efficiently endocytosed fluorescent nanoparticles.

Despite the fact that stimulation of macrophages with LPS in combination with IFN-γ leads to effective polarization of M1 macrophages and activation of their tumoricidal activity, these agents are unlikely to find practical use in humans due to their undesirable side effects. Bacterial endotoxin LPS is known to cause an uncontrolled immune response, endotoxic shock, splenomegaly, and tissue necrosis of immunocompetent organs [33, 34, 40]. Thus, the widespread use of bacterial lipopolysaccharides in medicine is largely excluded. In this regard, the natural triterpene glycoside, CA_2_-2, from sea cucumbers, characterized by desirable immunomodulatory properties at low nanomolar concentrations and the absence of harmful effects, may be a suitable inducer of macrophage polarization for cell immunotherapy of cancer.

One of the attractive and important strategies in cancer immunotherapy today is the repolarization of tumor-associated macrophages (TAM) of M2 phenotype into M1 phenotype macrophages. Currently, the molecular mechanisms that cause such macrophage reprogramming are being actively studied, and new methods are being developed for high-throughput screening of compounds that can cause macrophage reprogramming [41]. Holothurian triterpene glycoside cucumarioside A_2_-2 is a promising compound that promotes not only the activation of M0 macrophages and their polarization into the M1 phenotype but might also indirectly cause the repolarization of M2 macrophages at the concentration range of 10–50 nM that is much lower than its cytotoxic activity. The repolarization of macrophages from M2 to M1 phenotype in tumor tissue may be caused by M1-stimulated macrophages entering the tumor, actively producing TNF-α, ROS, and high iNOS/NO levels. An increase in their content in the tumor microenvironment (TME) can cause TAM reprogramming to the M1 phenotype. This, in turn, suppressed tumor growth due to the tumoricidal activity of reprogrammed macrophages [42]. Additionally, M1-polarized macrophages can recruit new Th1 cells via IFN-γ and chemokines CXCL9 and CXCL10 to kill tumor cells [43, 44].

Currently, the molecular mechanisms of the antitumor activity of macrophages are not fully understood. It is known that the inhibitory effect of M1 macrophages on tumor growth may be enhanced by the production of TNF-α, ROS, and nitric oxide. TNF-α plays a key role in inflammation and apoptosis of cancer cells through the tumor necrosis factor receptor 1 (TFNR1) signaling pathway [45]. TNF-α binds to the TFNR1 receptor triggering the conversion of Complex I to Complex II [46], in which Complex I is composed of TNFR1, tumor necrosis factor receptor type 1-associated DEATH domain protein (TRADD), receptor interacting protein 1 (RIP1), and TNF receptor-associated factor 2 (TRAF2) signals. Activated RIP1 binds to Fas-associated protein with death domain (FADD) and procaspase-8 and − 10 to form Complex II. Complex II induces ROS production and activation of caspase-3 and caspase-7 [47, 48]. TNF-α also induces cancer cell apoptosis through the MAPK-JNK pathway [47]. Thus, TNF-α is considered to be an effective anti-cancer cytokine [49, 50]. ROS and nitric oxide are signal molecules that play an important role in many physiological and pathological processes. Several studies have shown that ROS and NO generated by activated macrophages can kill different types of tumor cells by producing nitrosative/oxidative stress, inducing DNA damage, cytotoxicity, and apoptosis [51–54]. Therefore, ROS and nitric oxide generated in activated macrophages could also contribute to the anti-cancer effect. Based on the information and our own experimental results, it can be concluded that CA_2_-2 stimulates the polarization of M1 macrophages. The M1 macrophages showed accelerated migration towards tumor tissue, increased secretions of TNF-α, ROS, and NO, and enhanced cytotoxicity. Thus, ex vivo activation of macrophages by CA_2_-2 can serve as a useful technique for subsequent antitumor cellular immunotherapy.

The molecular mechanism of bacterial lipopolysaccharide action is fairly well-understood. In short, LPS binds the CD14/TLR4/MD2 receptor complex in many immune cells, which triggers the initiation of MyD88-dependent and MyD88-independent pathways leading to up-regulation of pro-inflammatory genes [55]. The molecular mechanism of CA_2_-2 immunomodulatory action is different. It has been proven [29] that the membrane molecular targets for CA_2_-2 are purinergic receptors of the P2X family (mainly P2X4 subtype) involved in the modulation of macrophage activity, synthesis and release of various cytokines, and the formation of inflammation, in phagocytosis, chemotaxis and initiation of apoptosis [56–58]. We do not exclude that it is this signaling pathway, mediated by modulation of the functional state of P2X4 receptors, leads to the activation and polarization of M1 macrophages and enhancement of their cytotoxic and antitumor effects.

## Conclusions

In summary, we found that the triterpene glycoside, cucumarioside A_2_-2, isolated from the holothurian *C*. *japonica*, is able to activate and polarize mouse macrophages toward M1 phenotype at nanomolar concentrations. This leads to a pronounced increase in the tumoricidal activity of macrophages against several types of tumor cells in vitro. M1 macrophages recognize and penetrate the tumor tissues much faster compared to M0 macrophages. This, in turn, leads to a marked increase in the number of M1 macrophages in the tumor tissues to suppress tumor growth and increase the average life span of tumor-bearing animals. Therefore, the use of the natural immunomodulator CA_2_-2 for ex vivo mouse macrophage activation and polarization can be a very useful approach for the subsequent development of antitumor cellular immunotherapy.

## Data Availability

The authors declare that all the data supporting the findings of this study are available within the article and its Supplementary Information files or from the corresponding author upon reasonable request.
